# The first fossil Coleoptera record from the Volyn Region, Ukraine, with description of a new *Glesoconomorphus* (Coleoptera, Mycteridae) in syninclusion with Winterschmidtiidae (Acari) and a key to species

**DOI:** 10.3897/zookeys.1068.75391

**Published:** 2021-11-08

**Authors:** Dmitry Telnov, Evgeny E. Perkovsky, Dmitry V. Vasilenko, Shûhei Yamamoto

**Affiliations:** 1 Department of Life Sciences, Natural History Museum, SW7 5BD, London, UK Natural History Museum London United Kingdom; 2 Institute of Biology, University of Latvia, O. Vācieša iela 4, LV-1004, Rīga, Latvia University of Latvia Rīga Latvia; 3 Coleopterological Research Center, Institute of Life Sciences and Technology, Daugavpils University, Vienības iela 13, LV-5401, Daugavpils, Latvia Daugavpils University Daugavpils Latvia; 4 I.I. Schmalhausen Institute of Zoology, National Academy of Sciences of Ukraine, B. Khmelnitskogo 15, 01601, Kiev, Ukraine National Academy of Sciences of Ukraine Kiev Ukraine; 5 A.A. Borissiak Paleontological Institute, Russian Academy of Sciences, Profsoyuznaya Str. 123, 117868, Moscow, Russia A.A. Borissiak Paleontological Institute, Russian Academy of Sciences Moscow Russia; 6 Cherepovets State University, Lunacharsky prospect 5, 162600, Cherepovets, Russia Cherepovets State University Cherepovets Russia; 7 Hokkaido University Museum, Hokkaido University, Kita 8, Nishi 5, Kita-ku, 060-0808, Sapporo, Japan Hokkaido University Sapporo Japan

**Keywords:** Eurypinae, identification, morphology, phoresy, Priabonian, Rovno amber, taxonomy

## Abstract

*Glesoconomorphusekaterinae***sp. nov.** (Coleoptera, Mycteridae), representing the first ever fossil species of Coleoptera from the Volyn Region of Ukraine and the first mycterid from late Eocene Rovno amber, is described and illustrated. A key to species of the fossil mycterid genus *Glesoconomorphus* Alekseev, Pollock & Bukejs, 2019 is presented. The systematic position of *Glesoconomorphus* within Eurypinae J. Thomson, 1860 is briefly discussed. The oldest finding of phoretic Winterschmidtiidae Oudemans, 1923 mites, found on the type specimen of the new beetle species, is reported.

## Introduction

*Glesoconomorphus* Alekseev, Pollock & Bukejs, 2019 was erected recently to hold the sole species *G.nachzehrer* Alekseev, Pollock & Bukejs, 2019 (Alekseev et al. 2019) from Eocene Baltic amber (Aleksandrova and Zaporozhets 2008; Iakovleva 2017; Iakovleva et al. 2021). The genus was attributed to Eurypinae J. Thomson, 1860 of Mycteridae Oken, 1843. Eurypinae is a well-defined subfamily within Mycteridae, with the following external features (Pollock 2010; Ivie and Pollock 2012): body slightly convex dorsally, head not produced to form a rostrum, compound eyes comparatively large, labrum exposed dorsally, insertions of antennae not concealed by lateral extension of frons, terminal maxillary palpomere subsecuriform, penultimate antennomere short, dorsal outline of pronotum subquadrate, pronotum laterally carinate, with paired postmedian impression, anterior margin of pronotum without transverse setal pad, scutellar shield flattened dorsally above level of elytra, elytra setose, irregularly punctate, procoxal cavities closed internally and open externally, procoxae contiguous, mesocoxal cavities closed internally and externally, penultimate tarsomere bilobate, tarsal claws swollen basally or with a distinct basal tooth (Alekseev et al. 2019). The male genital organs were not studied for *Glesoconomorphusnachzehrer* by Alekseev et al. (2019), and therefore their shape and structure remain unknown.

As discussed by Alekseev et al. (2019), *Glesoconomorphus* differs from other extant eurypine genera by the following combination of external features: ocular groove distinct, head without frontal furrows, compound eyes entire (non-emarginate) and strongly protruding from lateral outline of head, intrafacetal setae not present, pronotum not laterally carinate (this feature differs in the second species discussed below), frontoclypeal suture not indicated, dorsum evenly punctate and setose.

*Glesoconomorphus* appears close to the extant *Conomorphus* Champion, 1889 and *Stilpnonotus* Gray, 1832 due to the presence of a distinct ocular groove narrowly separated from the eyes (Pollock 2016; Alekseev et al. 2019). Among the fossil Eurypinae, *Glesoconomorphus* was reported to be different from *Bertinotus* Kirejtshuk & Nel, 2009 from Ypresian Oise amber due to the presence of paramedian depressions at the pronotal base, and a distinctly punctate head (Alekseev et al. 2019), and from *Europoeurypus* Alekseev, Bukejs & Pollock, 2020 in the absence of ocular grooves, the generally larger body (body length exceeding 10 mm), and the comparatively shorter antenna (Alekseev et al. 2020) (the length of the antenna should not be considered an important genus-rank feature in mycterids according to our expertise).

Rovno amber is considered the southern coeval of Baltic amber (Sokoloff et al. 2018), with extinct tropical elements supposedly represented better than in Baltic amber (Perkovsky 2013, 2016, 2017a, 2018; Colombo et al. 2021a; Radchenko and Perkovsky 2021). More than 300 arthropod species have been formally described from Rovno amber (authors’ data). The share of Baltic amber species from large arthropod orders and suborder reported from Rovno amber varies from less than 13% for Coleoptera species (Legalov et al. 2021a; Kupryjanowicz et al. 2021; Lyubarsky and Perkovsky 2021; Tshernyshev and Perkovsky 2021 and references therein) and 24% for Nematocera (Giłka et al. 2021) to less than 47% for Hymenoptera (Simutnik et al. 2020 and references therein; Colombo et al. 2021b; authors’ data). The new beetle discussed in the present paper is the fourth named fossil arthropod from amber from the Volyn Region of Ukraine. The first one was a new genus and species of cicadellid from Kovel (Dietrich and Perkovsky 2020), the second was a blattid from Manevichi (Anisyutkin and Perkovsky 2021), and the third was a new damselfly from Kovel (Martynov et al. in press).

The aims of the present paper are to describe and illustrate *Glesoconomorphusekaterinae* sp. nov., the first known Coleoptera species from Priabonian amber from the Volyn Region of Ukraine, to supplement the definition of the genus, and to provide a key to *Glesoconomorphus* species. The presence of an abdominal setal patch in *Glesoconomorphusekaterinae* sp. nov. confirms placement of the genus in the subfamily Eurypinae. The closure of the procoxal cavities and the presence of an apicoventral binding patch on each elytron, two other common features of Eurypinae (Pollock 2010), remain unknown in *Glesoconomorphus* since studying them was not possible in the available specimens.

## Materials and methods

Lisove is a new amber mine in the Volyn Region of NW Ukraine, 9 km east of Manevichi (the former Manevichi district, now Kamen-Kashirsky district).

Paired morphological structures are generally treated as singular in the text.

The specimen was studied and digital images were made using a Leica Z16 APO stereomicroscope equipped with Leica DFC450 Digital Camera at the I. I. Schmalhausen Institute of Zoology of the National Academy of Sciences of Ukraine (Kiev), and subsequently processed with the LAS Core 3.8 and Adobe Photoshop CS5 software.

The holotype is deposited at the Schmalhausen Institute of Zoology of the National Academy of Sciences of Ukraine, Kiev (**SIZK**).

## Results

### Order Coleoptera Linnaeus, 1758

#### Superfamily Tenebrionoidea Latreille, 1802

**Family Mycteridae Oken, 1843**

**Subfamily Eurypinae J.Thomson, 1860**

##### Genus *Glesoconomorphus* Alekseev, Pollock & Bukejs, 2019

###### Glesoconomorphus ekaterinae sp. nov.

Taxon classificationAnimaliaColeopteraMycteridae

327691F5-B01D-595B-9E8D-B614358EDF69

http://zoobank.org/30114DC4-AF1E-4143-B83E-232C1E53DE6A

Figs 1 , 2 , 3 , 4

####### Material examined.

***Holotype*** ♂ SIZK Les-10, Lisove, Volyn Region, Rovno amber, late Eocene // *Glesoconomorphusekaterinae* sp. nov. det. D.Telnov, E.Perkovsky, D.Vasilenko & S.Yamamoto, 2021 [printed]. Syninclusions are represented by three heteromorphic deuteronymphs of supposedly phoretic Winterschmidtiidae Oudemans, 1923 mites attached to abdominal ventrites III, IV, and V of the beetle.

####### Type locality.

Lisove, Volyn Region, NW Ukraine.

####### Derivatio nominis.

Patronymic. The new species is named in honour of Ekaterina A. Sidorchuk (1981–2019), who was a renowned acarologist and our helpful colleague.

####### Measurements.

***Holotype*** ♂, total body length 2.95 mm; head length 0.29 mm, head width across compound eyes 0.67 mm, pronotal length 0.59 mm, maximum pronotal width 0.66 mm, elytral length 2.17 mm, combined maximum elytral width across postmedian area 1.15 mm.

**Figure 1.**
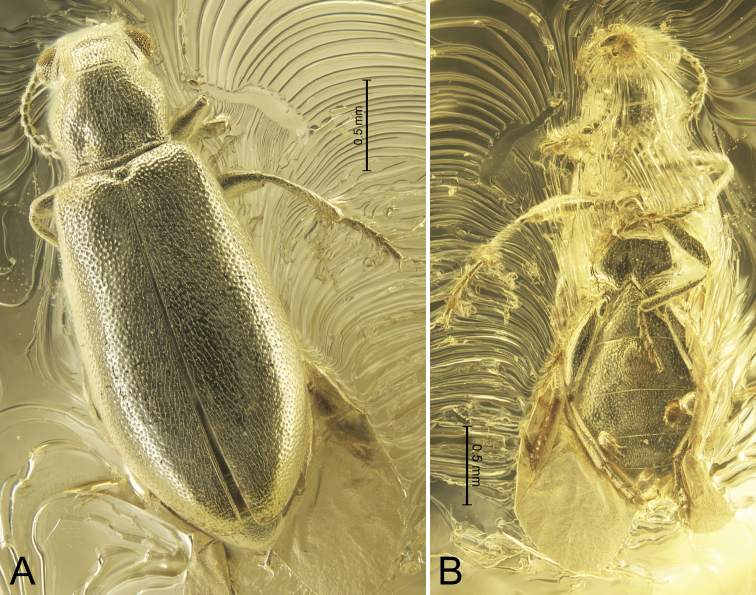
*Glesoconomorphusekaterinae* sp. nov., holotype ♂ **A** dorsal habitus **B** ventral habitus.

####### Description.

♂, body cylindrical, slightly convex in dorsal aspect. Dorsum and venter uniformly dark brown with weak metallic coppery lustre, compound eyes reddish brown. Head flattened dorsally, glossy. Labrum transverse, moderately densely punctate, anterior margin broadly emarginate. Frontoclypeal suture not present. Anterolateral margin of frons slightly prominent laterodorsad, not concealing insertion of antenna. Broad, shallow paired frontal furrow present. Minimum interocular distance 1.6× the dorsal eye length. Ocular (suborbital) groove present, distinct, traceable from frontal canthus to posterolateral extent of compound eye, narrowly separated from eye in anterior part, becoming more distant from it in posterior part. Compound eye large, entire, hemispherical, strongly prominent laterally in dorsal view. Interfacetal setae not present. Tempora slightly constricted posteriad, 0.34× dorsal eye length. Head dorsal punctures circular to slightly elliptical, rather large, moderately deep and dense. Intervening spaces glossy, glabrous, on frons generally as wide as to 1.5× wider than punctures. Punctures generally smaller on head base. Inconspicuous, appressed, anteriad-directed setae rising from centre of each puncture, generally not or slightly surpassing length of adjoining puncture. Antenna moniliform, rather short, in male extending towards base of pronotum. Basal antennomere cylindrical, about 1.1× longer than antennomere two. Antennomere three of about same length as preceding antennomere. Antennomeres 8–10 subtriangular, widened distally. Antennomeres 9–10 slightly transverse. Terminal antennomere elliptical, about 1.2× longer than penultimate antennomere. Terminal maxillary palpomere subsecuriform. Pronotum flattened dorsally, widest in anterior half, slightly constricted laterally towards base. Anterior and posterior margins truncate to subtruncate. Anterior bead not observed, posterior bead well-defined, broad. Anterolateral angles obsolete, broadly rounded. Posterolateral angles obtuse angulate. Lateral margin of pronotum modified into an obtuse carina delimiting pronotal disc from pronotal hypomeron (observed in lateral view). Pronotal disc with paired, moderately large, shallow, longitudinal, elliptical postmedian impression. Pronotum densely and roughly punctate dorsally and laterally, punctures nearly circular. Intervening spaces glossy, glabrous, generally narrower than punctures except in median part of pronotal disc, where intervening spaces are from as large as to twice as large as punctures. Dorsal pronotal setae similar to those on head. Scutellar shield small, widened posteriad, subtruncate at posterior margin, roughly punctate. Elytron moderately strongly elongate, slightly widened in apical third, slightly convex dorsally. Humerus broadly rounded. Humeral callosity not present. Postbasal transverse impression not indicated. Apical sutural angle broadly rounded. Epipleura rather broad in basal half of elytra. Sutural stria narrow, present in apical fourth of elytra. Elytral punctures rather large, deep and dense, intervening spaces glossy and glabrous, from as large as to narrower than punctures. Each puncture with an inconspicuous, appressed, moderately long, posteriad-directed seta. Metathoracic wings fully developed (functional), long, visible veins darkly pigmented. Radial cell present, fully closed. Legs moderately long, rather robust, finely and sparsely pubescent. Femora thickened but not clavate, glossy, sparsely punctate, subequal in length to tibiae. Ventral side of tarsomeres densely setose. Male basal metatarsomere 1.5× shorter than combined length of remaining metatarsomeres. Metasternum slightly convex in ventral aspect, glossy, sparsely punctate. Abdominal ventrites rather densely and roughly punctured laterally. Intercoxal process of abdominal ventrite I with particularly large and dense, shallow punctures. Male abdominal ventrite II modified, with an elliptically grouped median group of dense, elongate, scale-like setae comprising a sex patch. Setae of sex patch reaching neither anterior nor posterior margin of male abdominal ventrite II. Male tergite VII broadly rounded at posterior margin, densely punctate-corrugate dorsally. Male genital organs and remaining terminalia not exposed and not studied.

**Figure 2.**
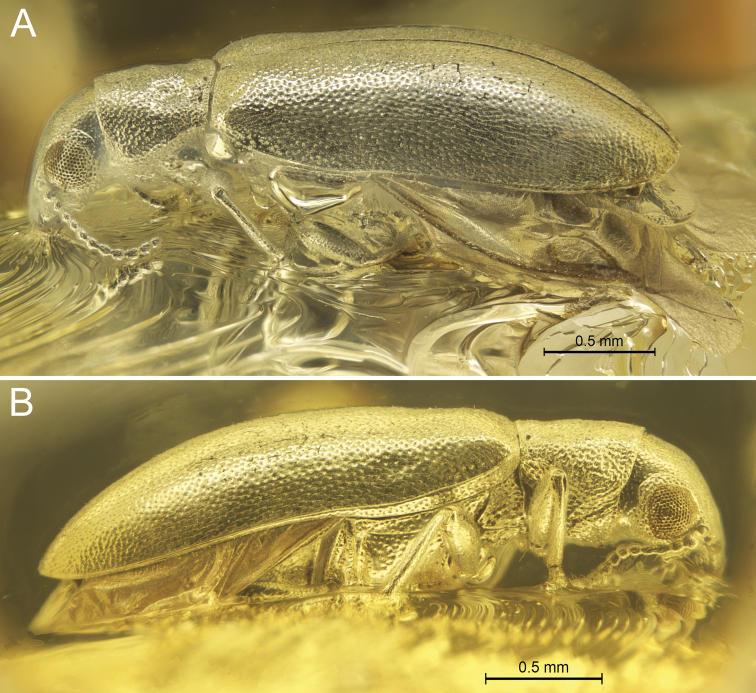
*Glesoconomorphusekaterinae* sp. nov., holotype ♂ **A** left lateral habitus **B** right lateral habitus.

####### Sexual dimorphism.

Female unknown.

####### Differential diagnosis.

The new species is generally close to *G.nachzehrer* but specifically different in the comparatively less slender body, the dorsal outline of the pronotum, which is constricted towards the base (lateral margins of pronotum subparallel in *G.nachzehrer*), the transverse pronotum (pronotum subquadrate to slightly wider than broad in *G.nachzehrer*), the pronotal disc delimited from the pronotal hypomeron by an obtuse carinate lateral margin in lateral view (lateral margin of pronotum not carinate in lateral view in *G.nachzehrer*), the presence of frontal furrows (not observed in *G.nachzehrer*), the comparatively less densely punctured frons with some of the intervening spaces twice as wide as the generally circular to slightly elliptical punctures (frons somewhat denser punctured, with punctures generally elongate, in *G.nachzehrer*), the labrum broadly emarginate at the anterior margin (labrum broadly rounded in *G.nachzehrer*), the comparatively stronger elytral punctures (punctures on elytra comparatively smaller in *G.nachzehrer*). The holotype of *G.ekaterinae* sp. nov. is also significantly smaller than the holotype of *G.nachzehrer* (total body length 2.95 mm *vs* 3.6 mm), which should not be considered as an important comparative feature. Indeed, the males of some Eurypinae (for instance, *Omineus* Lewis, 1895) are known to be smaller than the females (Alekseev et al. 2020). We consider the holotype of *G.nachzehrer* a female (this was not stated by the authors of this taxon, see Alekseev et al. (2019)), therefore the difference in body length between the two species should not be considered very significant until the discovery of male *G.nachzehrer*.

**Figure 3.**
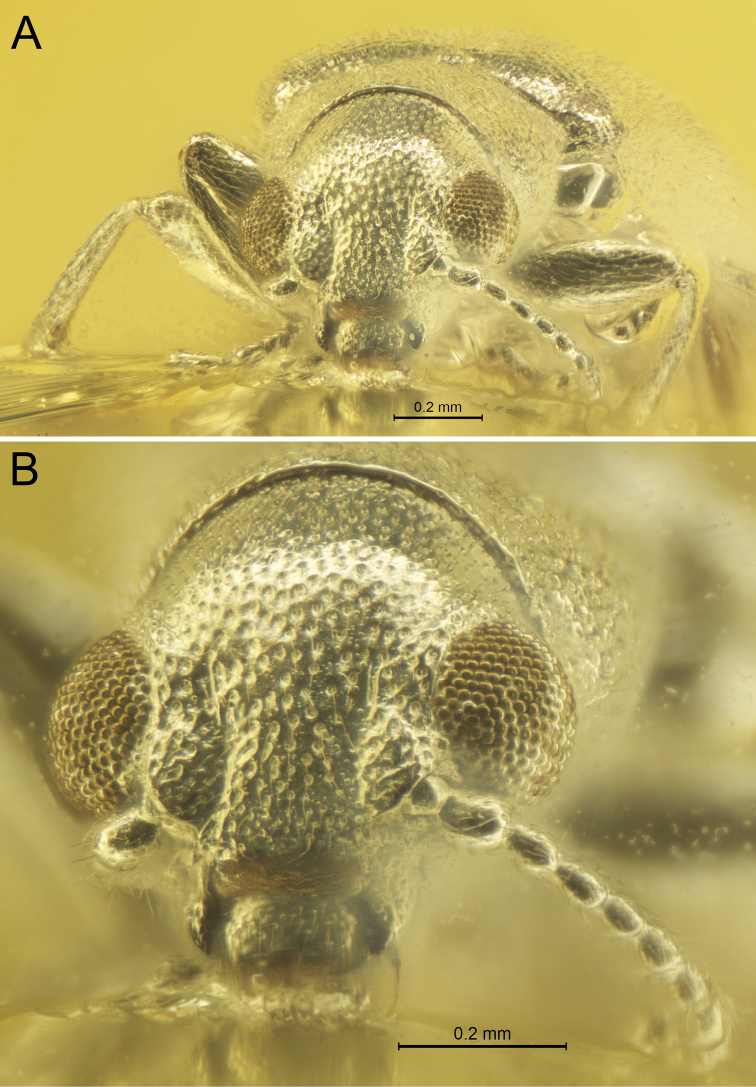
*Glesoconomorphusekaterinae* sp. nov., holotype ♂ **A** frontal habitus **B** head in frontal view.

**Figure 4.**
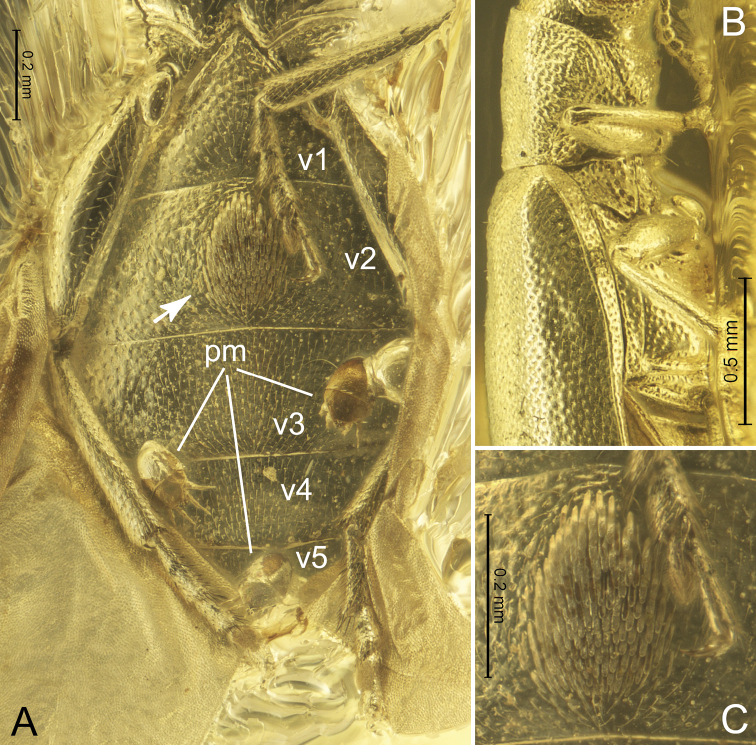
*Glesoconomorphusekaterinae* sp. nov., holotype ♂ **A** abdomen in ventral view (arrow indicates a setal patch) **B** pro-, meso-, and metathorax in right lateral view **C** sexual setal patch on ventrite II, enlarged. Abbreviations: pm = phoretic mite (Acari: Winterschmidtiidae), v1–v5 = ventrites I-V.

### Key to species of *Glesoconomorphus*

The present key excludes sexual features, since both sexes remain unknown for each of the two known species.

**Table:** 

1	Pronotum distinctly transverse, lateral margins broadly rounded, constricted prebasally; pronotal disc delimited from hypomeron by obtuse carinate lateral margin (lateral view); frontal furrows present; ratio of elytral length to combined width of elytra 1.89; labrum broadly emarginate at anterior margin	***G.ekaterinae* sp. nov.**
–	Pronotum nearly as long as wide, lateral margins subparallel, not or vaguely constricted prebasally; transition of pronotal disc to hypomeron even, lateral margin of pronotum not carinate in lateral view; ratio of elytral length to combined width of elytra 2.12; labrum broadly rounded at anterior margin	***G.nachzehrer* Alekseev, Pollock & Bukejs, 2019**

## Discussion

The composition of fossil Mycteridae was discussed recently in Alekseev et al. (2019, 2020) and is therefore not repeated here. The fact that *Conomorphus* Champion, 1889 and *Stilpnonotus* Gray, 1832, the two thermophilic mycterid genera nearest to *Glesoconomorphus*, are nowadays restricted to the Neotropics (Pollock 2016) indicates that *Glesoconomorphus* possibly was more common in Rovno amber forest than in Baltic amber forest (Mänd et al. 2018).

The presence of a sex patch on abdominal ventrite II in male *Glesoconomorphusekaterinae* sp. nov. and the presence of distinct frontal furrows provide additional support for the placement of *Glesoconomorphus* in the subfamily Eurypinae of Mycteridae. The structure of the procoxal cavities and the presence or absence of an apicoventral binding patch on each elytron remain unknown in this genus.

Nearly all studied Rovno amber inclusions from the Rovno Region (reported inclusions from Zhitomir Region listed in Melnitsky et al. 2021a) were collected from Klesov and from the Horyn’ River Basin (Perkovsky et al. 2010; Perkovsky 2017b; Mitov et al. 2021); however, new material has been collected from the Varash district (the former districts of Vladimirets and Zarechnoye) of the Rovno Region and from the former Manevichi district of the Volyn Region (basins of the Styr, Veselukha, and Stokhod rivers). These new collections (mostly from Kuchotskaya Volya, Voronki, and Velyki Telkovichi) have revealed many new taxa of Dictyoptera (including Isoptera), Coleoptera, Hemiptera, Hymenoptera, Neuroptera, Raphidioptera, and Trichoptera, as listed in Tshernyshev and Perkovsky (2021), with additions in Legalov et al. (2019, 2021b), Matalin et al. (2021), Giłka et al. (2021), Melnitsky et al. (2021b), Jałoszyński and Perkovsky (2021), and Golub et al. (2021). The new locality (Lisove) and the locality Manevichi belong to the Styr River Basin and could be the source of important new findings, e.g., the hitherto oldest published record of Winterschmidtiidae was known from Miocene Mexican amber (Klimov et al. 2021).

The type species of *Glesoconomorphus* from Baltic amber is named after “Nachzehrer”, a mystical vampire from the folklore of Germany and Poland that, according to the legend, first persecutes and then murders its natural relatives (Alekseev et al. 2019). We can only speculate that individuals of the type species could not cross the ancient sea and reach the Volyn area (Ivanov et al. 2016, fig.1), where *Glesoconomorphusekaterinae* sp. nov., the first of the Rovno amber mycterids, dwelled.

## Supplementary Material

XML Treatment for Glesoconomorphus ekaterinae
